# Autogenous fibula graft and cannulated screw fixation to cephalic cut out after DHS fixation: a retrospective study

**DOI:** 10.1186/s13018-019-1521-2

**Published:** 2020-01-15

**Authors:** Yan Sun, Tao Huang, Jiangtao Lin, Junbo Ge, Benjun Bi, Zhilin Cao, Huanyu Hong

**Affiliations:** Department of Orthopedics, Yantaishan Hospital, Yantai, China

**Keywords:** Atogenous fibula graft, Dynamic hip screw plate, Femoral head cutting, Intertrochanteric fracture

## Abstract

**Background:**

This study aimed to explore the effect of the treatment through autologous fibula graft and hollow needle fixation to treat femoral head cutting after dynamic hip screw (DHS) fixation.

**Methods:**

A total of 41 patients were admitted to the department of orthopedic trauma and received DHS fixation. Preoperative and postoperative harris score of hip function, limb shortening length and collodiaphysial angle between operation group (*n* = 11) and non-operation group (*n* = 13) were compared.

**Results:**

There was no difference between the two groups before surgery (*P* > 0.05). There was a difference between the preoperative and postoperative in the operation group (*P* < 0.05). The excellent and good rate of the hip function score in patients 6 months after the operation was 55.6%. In the operation group, the hip function score increased after surgery (*P* < 0.001). Except for two groups of patients before operation, there was a difference in the limb shortening length and collodiaphysial angle between the operation group and non-operation group in other time points after surgery (*P* < 0.001).

**Conclusion:**

The application of the autogenous fibula graft and hollow nail fixation was effective in treating femoral head cutting after DHS fixation, and patients’ subjective evaluation and objective indicators’ outcomes of follow up were satisfactory, which was worthy of clinical application.

## Background

Dynamic hip screw fixation (DHS), or sliding hip screw fixation, has been the most common internal fixation for intertrochanteric fractures (ITF). The system has been first designed by Pohl in 1951 and applied by Schumpelik in 1955 [1]. Since 1970, it has been widely used globally and was refined by the Swiss Society of Internal Fixation later, with a short abbreviation as DHS. At present, DHS has been recognized as the gold standard for stable ITF treatment, with features such as firm fixation, easy-to-perform and confirmative efficiency [2–4]. As its wide application, some accompanying failures such as femoral head cutting, fracture translocation due to too much hip screw sliding, fracture unhealing, and injured limb shortening [5, 6].

**Fig. 1:**
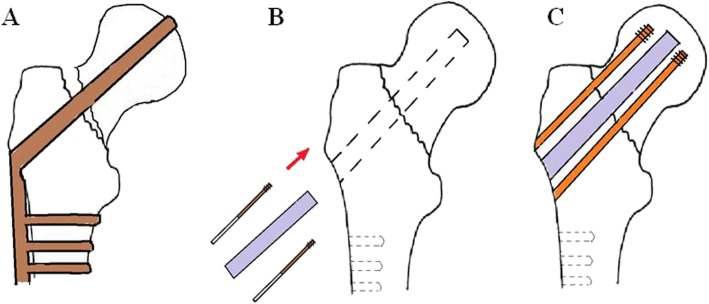
**a** DHS fixation of femoral intertrochanteric fracture failed. DHS screws appear cut and the nail tail protrudes from the femoral head. The length of the limb becomes shorten and the neck dry angle smaller. **b** Autogenous fibula graft and cannulated screw fixation to cephalic cut out after DHS Fixation. Fracture reduction will be performed after DHS is removed. Measure and correct the required fibula length to match the diameter of the remaining nail canal. **c** Femoral intertrochanteric fracture reduced again. Hip varus recovers well and the hollow nails are in a good position

The treatment of coxa varus deformity due to femoral head cutting after DHS fixation has been a difficult clinical problem. A total of 1685 patients with ITF were admitted to the Department of Orthopedic Trauma of our hospital and received DHS fixation from March 2007 to March 2015. In these patients, 41 cases had femoral head cutting, 11 of whom had autogenous fibula graft plus hollow nail fixation treatment and had satisfactory results. We retrospectively investigated the effect of autogenous fibula graft and hollow needle fixation in treating the femoral head cutting after the DHS fixation and reported as follows (Fig. 1).

## Methods

### Participants

**Fig. 2:**
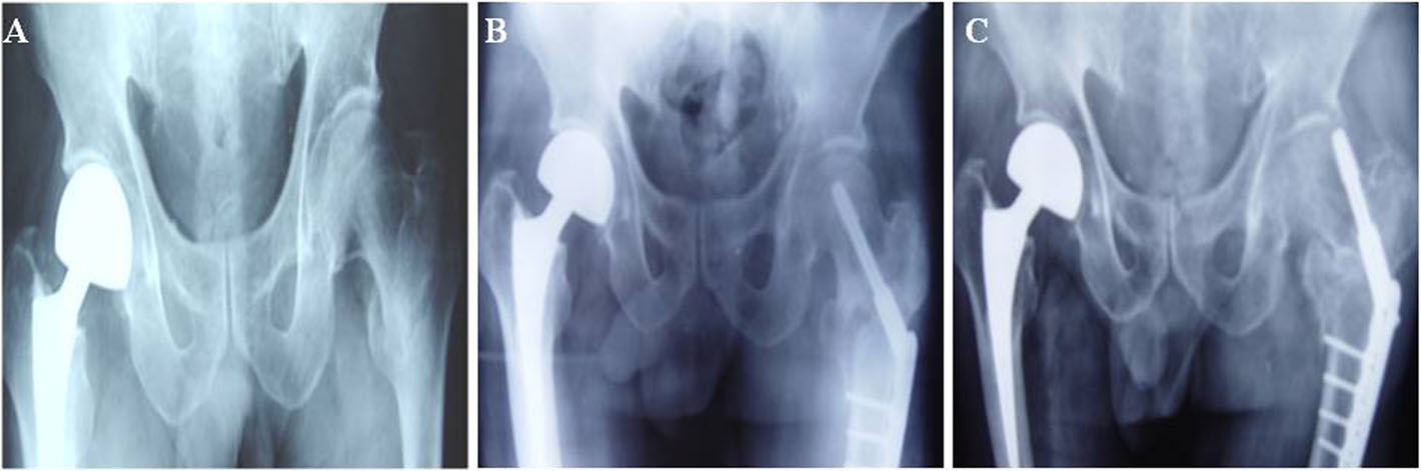
Patient, male, 68 years old, had a history of right femoral neck fracture with parallel femoral head replacement. **a** 1 day after the fall, he was admitted to the hospital. The left femur intertrochanteric fracture, the left lower extremity was shortened by 3 cm. **b** The left side DHS fixation was performed 5 days after admission, and the fracture reduction was slightly worse. **c** 142 days after DHS fixation, left hip varus deformity, limb shortening, DNS screw cutting, nail tail protruding femoral head

A total of 1685 patients with ITF were admitted to the Department of Orthopedic Trauma of our hospital and received DHS fixation from March 2007 to March 2015. There were 739 male patients and 946 female ones, aged between 23 and 96 years old (mean 70.1 years), and their clinical data were retrospectively collected. Operations were conducted in strict accordance with standard DHS fixation operation method, and the location of internal fixation was affirmed under the C arm fluoroscopy. After the operation, patients could walk with crutches (4 weeks later), had weight-bearing exercise (8 weeks later) (Fig. 2). They were followed up for 6 to 24 months, and their X-ray films at preoperative, postoperative, and follow-up periods were collected. Among the patients, 9.44% patients had internal fixation failure (159/1685 cases), and all patients suffered different levels of the hip joint pain, limb shortening, lameness, and limitation of joint activities, etc. in the injured side. In those failure cases, there were 41 cases had femoral head cutting (2.43%) during DHS fixation, from whom 11 patients took autogenous fibula graft plus hollow nail fixation treatment, 17 patients treated with an artificial hip replacement, and 13 patients gave up treatment.

In the autogenous fibula graft and hollow nail fixation operation group, there were 11 patients including 4 male patients and 7 female ones, aged between 43 to 78 years old (mean 67.2 years). There were ITF on the left side (3 cases) and the right side (8 cases). According to the Evans classification [7], the cases could be divided into type I (0 case), type II (2 cases), type III (3 cases), type IV (4 cases), and type V (2 cases). While according to the Singh classification [8], their osteoporosis condition could be classified as level I (6 cases), level II (1 case), level III (2 cases), level IV (1 case), and level V (1 case). There were different causes for the injury, including traffic accident injury (2 cases), high falling injury (1 case), and being hurt when walk or bike riding (8 cases). Moreover, the patients accompanied with some chronic diseases, such as high blood pressure (10 cases), heart disease (6 cases), diabetes (3 cases), pulmonary insufficiency (2 cases), and sequelae of cerebral embolism (2 cases). The clinical data table of operation group and non-operation groups of patients were seen in Table 1.

**Table 1:** Clinical data table of two groups of patients (*n* = 24)

Indicators	Types	Harris score of hip joint function							
		Operation group (*n* = 11)				Non-operation group (*n* = 13)			
		> 70	≤ 70	*χ2*	*P*	> 70	≤ 70	*χ2*	*P*
Sex	Male	0	4	0.629	0.428	1	4	0.043	0.835
	Female	1	6			2	6		
Parts	Left	1	2	2.933	0.087	2	4	0.660	0.416
	Right	0	8			1	6		
Evans classification	I	0	0	3.091	0.636	0	0	3.780	0.427
	II	0	2			1	0		
	III	1	2			1	2		
	IV	0	4			1	5		
	V	0	2			0	3		
Singh classification	I	0	6	5.944	0.455	1	4	0.706	1.000
	II	0	1			1	4		
	III	1	1			1	2		
	IV	0	1			0	0		
	V	0	1			0	0		
Cause of injury	Low energy	1	8	0.244	0.621	2	10	3.611	0.057
	High energy	0	2			1	0		
DNS fixed point	Upper 1/3	0	4	1.861	1.000	0	2	1.376	0.738
	Middle 1/3	0	3			1	5		
	Lower 1/3	1	3			2	3		
Nail tail position	Not out of the femoral head	1	3	1.925	0.165	0	8	6.240	0.012
	Out of the femoral head	0	7			3	2		

After the DHS fixation operation, some patients readmitted to the hospital in 95.3 days (59–137 days) due to hip pain, and various degrees of coxa varus deformity with limb shortening from 0.9 to 3.6 cm (mean 2.45 cm). In these patients, they had DNS screw been fixed at the upper 1/3 (4 cases), middle 1/3 (3 cases), and lower 1/3 (4 cases) of their femoral neck. The distance from the end of DNS screw to the top tip of the femoral head was between 1.0 and 2.8 cm (mean 1.78 cm), and there were 4 cases with a screw not penetrating the femoral head, and 7 ones with screw penetrating the femoral head.

### Pre-operative preparations

All patients were hospitalized for 3 to 7 days before surgery (mean 3.80 days). Their operation tolerance was comprehensively evaluated, and those patients with hypertension, heart disease, diabetes, and lung dysfunction received antihypertensive, hypolipidemic, hypoglycemic therapy, etc. to control their blood pressure to be less than 150/90 mmHg and blood glucose lower than 8 mmol/L after consultation with the department of internal medicine. Each preoperative examination was completed and heart arrhythmia was controlled. Anemia, water, and electrolyte disorder, as well as acid and alkali imbalance, were corrected. Those patients accompanied with cardiovascular diseases received ECG monitoring during and after surgery. Antibiotics were administered to prevent infection in 30 min before surgery.

### Surgical approach

General or epidural anesthesia was performed. The patient was in a supine position on a traction bed under the guidance of C-arm X-ray fluoroscopy. A longitudinal incision was made at the skin of lateral thigh at the great trochanter site, then to the one-third of the posterior greater trochanter via the top tip of the greater trochanter and moved down about 10 to 15 cm long. Skin, subcutaneous tissue, the deep fascia, and tensor fascia was cut in sequence to expose the vastus lateralis muscle. The bone of the upper segment of the lateral femur was exposed via the posterior vastus lateralis muscle. After DHS screw was completely removed, tissues along anterior and posterior femoral trochanter were separated until the fracture site and the connective tissue around which was removed. Manual relocation of fracture end was made after tissue release, and part of the middle gluteal muscle and muscle group involved in the internal rotation could be dissected when difficulty occurred during the relocation. When the relocation was satisfied, two Kirschner needle (size 2.5 mm) was inserted to fix the fracture. To measure the length of the fibula needed for the cavity from the site of the needle into femur trochanter to the femoral head. The middle ipsilateral fibula was obtained and its diameter was pruned to match the size of the needle pathway, ensuring their close contact. The position of the Kirschner needle was adjusted to form a triangle to the fibula. Then, drilling was made and two hollow nails (size 7.3 mm) were screwed inside, the tail of which was about 1 cm distance from the tip of the femur. After confirmation of X-ray fluoroscopy, the incision was washed, meanwhile, hemostasis was made and a negative pressure drainage tube was placed. The incision was sutured layer by layer and closed finally.

### Fibula obtainment

An incision (10–12 cm) was made on the lateral side of the up-middle part of the leg, extending downward (3–4 cm below the fibular head) along the longitudinal axis of the fibula. The deep fascia was exposed to the fibula from the muscle gap between the musculus peroneus brevis and longus and soleus. The lateral side of the fibula was exposed after the separation of the musculus peroneus brevis and longus and the surrounding fascia. The starting points of the musculus peroneus brevis and longus were separated and a little extra periosteal tissue was preserved carefully. The partial fibula (8–10 cm) was cut (5–7 cm under the fibular head), and its cutting length was determined to meet the need for measurements with a navigator. After the truncation of the fibula from the proximal and distal end, then the fibula is pulled outside, and the attachment point of the musculus extensor digitorum to the fibula was separated to expose the interosseous membrane. Then, the interosseous membrane was cut off to expose the peroneal vessel. The peroneal vessel was cut and its distal part was ligated. The fibula was pulled anteromedially, and the attachment of the flexor digitorum longus on the fibula was removed. Then the fibula was washed with saline and wrapped with a gauze pad soaked in the saltwater for further usage.

### Post-operative treatment

Antibiotics were administered for 2–3 days after the surgery to prevent infection and regulate the water and electrolyte balance. The distal end of the injured limb was elevated after the surgery and the negative pressure drainage tube was removed after 48–72 h. The dressing was changed in regular and the suture of incisions was removed 14 days later. The limb was in an abduct neutral position or received transcutaneous traction and fixation quadriceps for 2 weeks, and then the femoris received active static contraction exercises. Patients had CPM functional training after 4 weeks. X-ray examination in the 12th week after surgery suggested forming a callus, and patients could walk with crutches and do some weight-bearing activities. Half of a year later, X-ray recheck showed fracture healing and patients could walk without crutches.

### Observation and efficiency evaluation criteria

General operative conditions including the operation time, the amount of intraoperative bleeding, and the length of operative incision were recorded. Post-operative recheck was administered regularly, and fracture’s healing time was recorded. The efficiency was evaluated based on the X-ray and the Harris score [9] of hip joint function in 1, 3, and 6 months after the operation. Harris score was calculated in terms of pain, function, deformity, and bone joint activity: (1) excellent when the score was between 91 and 100. The fracture is healed completely, with no pain in the hip and recovered bone joint functions; (2) good when the score was between 81 and 90. The fracture is healing, with hip pain occasionally and mostly recovered bone joint functions; (3) middle when the score was between 71 and 80. The fracture is healing, with hip pain sometimes, hip varus, and limited bone joint function is (4) poor when the score was under 70 points, the fracture is malformed or unhealed, with hip pain, and disability of walking.

### Statistical analysis

All statistical analyses were performed with SPSS software (version 17.0). Numeric data was represented by the number of examples, and *χ2* or Fisher’s test was used for comparison between groups. While measurement data was represented as *x* ± *s*, and independent sample *t* test was conducted for variables’ comparison. Repeated measurement data analysis of variance was performed to compare those variables at different time points. α value equal to 0.05 was treated as the test level, and *P* value less than or equal to 0.05 was regarded as a statistically significant difference.

## Results

The length of hospital stay of 11 patients in the operation group ranged from 9 to 18 days (mean 13.6 days). The hip operation and the fibula resection were performed at the same time. The operation time was between 75 and 145 min (mean 98.2 min). The incision length of hip operation was between 11 and 15 cm (mean 12.7 cm). The amount of dominant operating bleeding was between 260 and 400 ml (mean 345.5 ml). There was no bleeding transfusion, and no death case due to surgery. Post-operative X-ray test showed that fracture replacement and internal fixation were well. All patients were followed up for 14 to 28 months (mean 19 months). No complications such as incision infection, pneumonia, and deep venous thrombus occurred. Two patients died from cardiocerebral and pulmonary diseases in 2 and 5 months after the operation. All patients had clinical healing time for 6 to 11 months (mean 8.1 months), with no screw breakage. There was one case with screw loosening and withdrawal, nine cases with complete healing, two cases with incomplete healing, and one case with femoral head necrosis. The Harris score of hip function in nine patients 6 months after the operation was excellent in one case, good in four cases, middle in three cases, poor in one case, and the excellent and good rate was 55.6%. In the operation group, the hip function score increased significantly after surgery more than that score before surgery, with a statistically significant difference. The comparison of hip function score between two groups of patients before and 6 months after operation were seen in Table 2.

**Table 2:** The comparison of hip function score between two groups of patients before and 6 months after operation (*n* = 21)

Groups	Harris score of hip joint function	Before the operation	6 months after the operation	*χ2*	*P*
Operation group (*n* = 9)	> 70	1	7	4.197	0.040
	≤70	8	2		
Non-operation group (*n* = 12)	> 70	3	1	1.200	0.273
	≤ 70	9	11		

The comparison of pre-operative and post-operative limb shortening length and the collodiaphysial angle between the two groups were seen in Table 3. There was no significant difference in the limb shortening length and collodiaphysial angle between the operation group and the non-operation group (*P* > 0.05). There was a significant difference between the preoperative and postoperative limb shortening length and collodiaphysial angle before and after surgery. There was an interactive effect between the preoperative and postoperative time and groups.

**Table 3:** Comparison of the length of limb shortening and neck dry angle between the two groups of patients before and after the operation (*n* = 24)

Groups	Indicators	Before the operation (^−^*x* ± *s*)	After the operation (^−^*x* ± *s*)	Sum	*F*	*P*
Operation group (*n* = 11)	Length of limb shortening neck dry angle	2.54 ± 0.74 101.8 ± 7.74	1.23 ± 0.50 122.0 ± 5.04	1.88 ± 0.91 111.9 ± 12.14	23.425 52.486	0.000 0.000
Non-operation group (*n* = 13)	Length of limb shortening neck dry angle	2.15 ± 0.72 105.9 ± 8.05	2.15 ± 0.72 105.9 ± 8.05	2.15 ± 0.70 105.8 ± 7.89	0.000 0.000	1.000 1.000
Sum (*n* = 24)	Length of limb shortening neck dry angle	2.33 ± 0.74 104.0 ± 8.01	1.73 ± 0.77 113.3 ± 10.60		73.674 114.662	0.000 0.000
*t*	Length of limb shortening neck dry angle	1.303 1.243	3.564 5.981	0.967 4.428	*F* = 7374 *F* = 114.662	
*P*	Length of limb shortening neck dry angle	0.206 0.227	0.002 0.000	0.336 0.047	*P* = 0.000 *P* = 0.000	

**Fig. 3:**
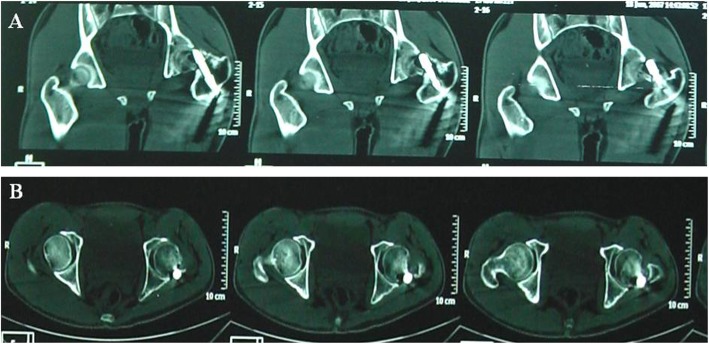
Patient, male, 72 years old, left femoral intertrochanteric fracture, 109 days after DHS fixation. CT scan showed left hip varus deformity and limb shortening. DNS screw appeared to cut and nail tail protruded femoral head. **a** hip CT coronal position, **b** hip joint CT level

**Fig. 4:**
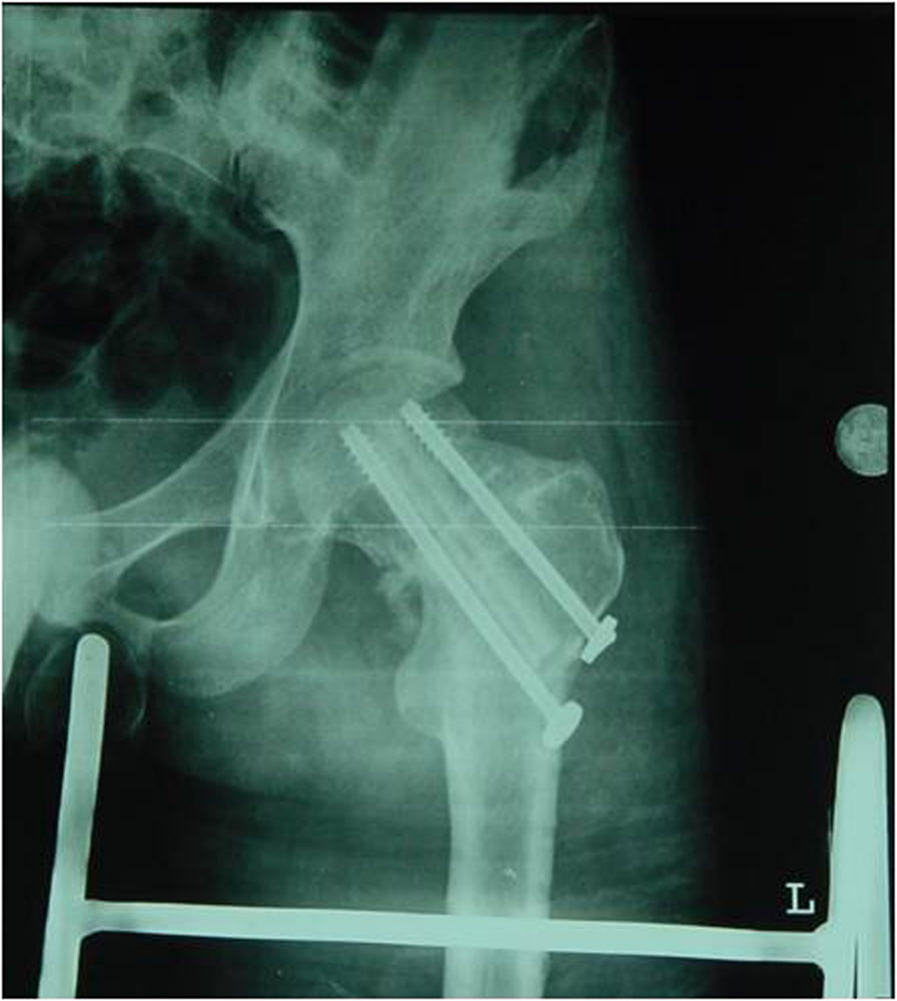
One week after autologous iliac bone graft and cannulated screw fixation. The left hip varus was well reset and the position of the cannulated nail was good. The transplanted humerus (the hip X-ray anterior piece) was seen in the bone channel after the DNS screw was removed

**Fig. 5:**
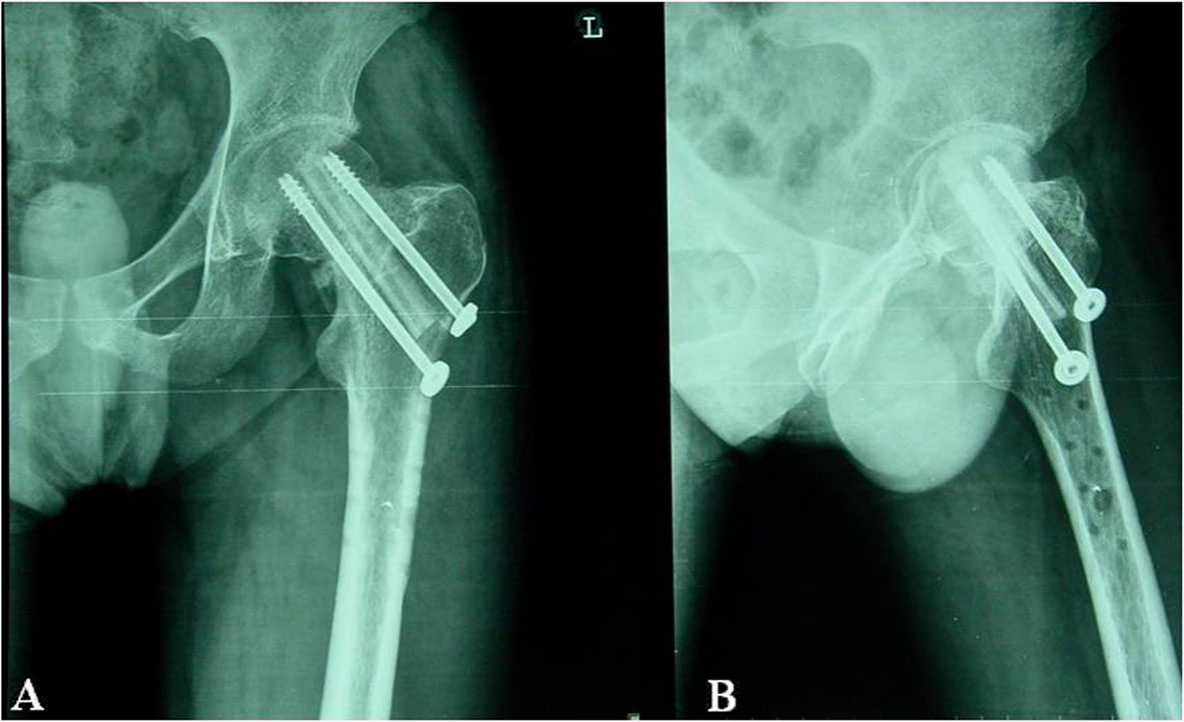
Three months after autologous iliac bone graft and cannulated screw fixation. The left hip varus was well restored, the position of the cannulated nail was good, the fracture line was blurred and the humeral bone shadow was observed. **a** hip X-ray anterior piece, **b** hip joint X-ray lateral slice

**Fig. 6:**
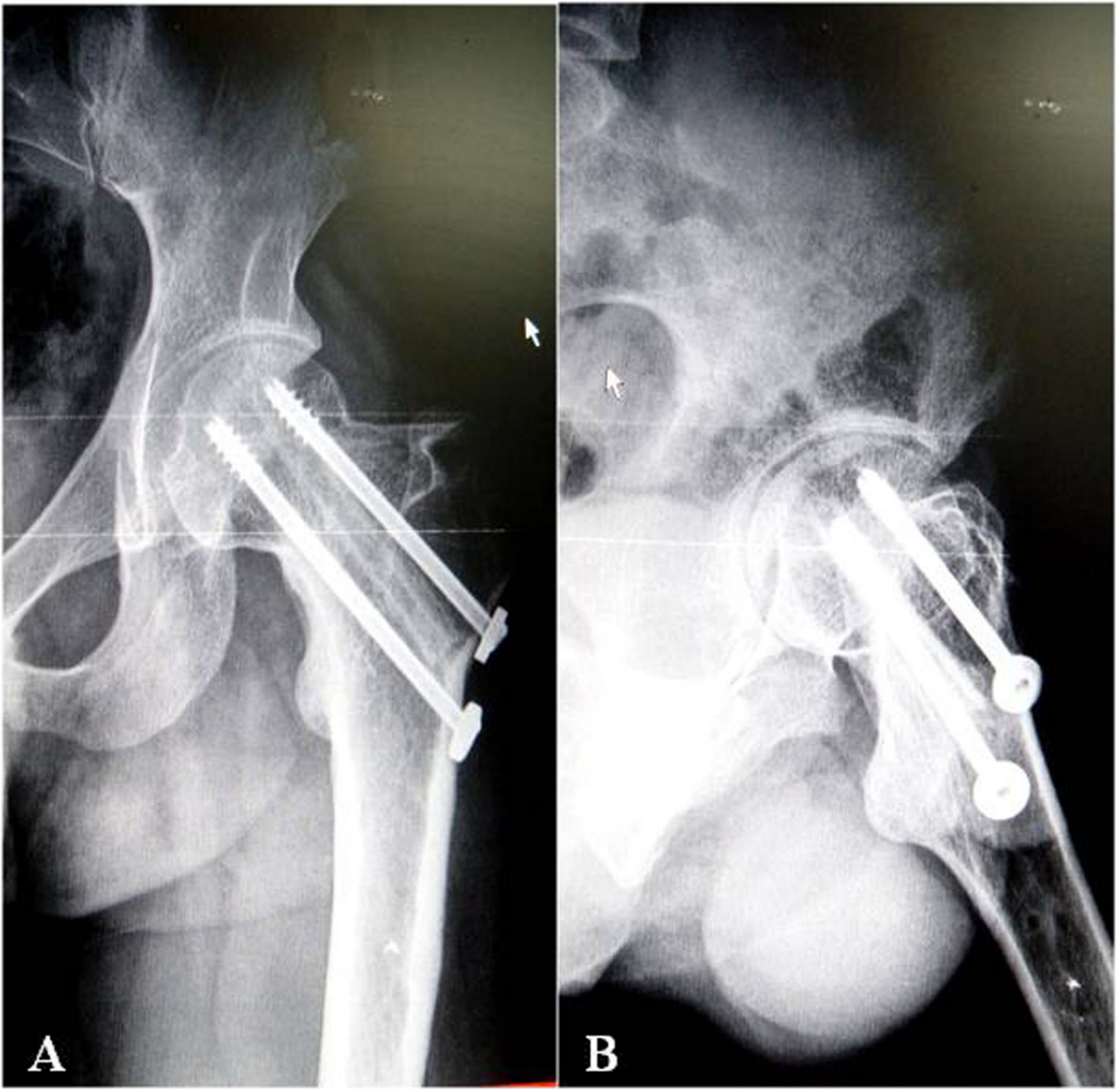
Six months after autologous iliac bone graft plus cannulated screw fixation, left hip joint reduction, cannulated nail fixation, blurred fracture line, blurred humeral bone, and good fracture healing. **a** hip X-ray anterior piece, **b** hip joint X-ray lateral slice

Preoperative and postoperative limb shortening length and collodiaphysial angle between two groups were compared and the results were shown in Table 4. There was no significant difference in the limb shortening length and collodiaphysial angle between the operation group and non-operation group. There was a significant difference between the preoperative and postoperative limb shortening length and collodiaphysial angle. There was an interaction effect between the preoperative and postoperative time and groups (Figs. 3, 4, 5, and 6).

**Table 4:** The length of limb shortening and the neck dry angle of the patients in the 3 months after operation were compared with those in the non-operation group (*n* = 22)

Groups	Indicators	Before the operation (^−^x ± s)	After the operation (^−^x ± s)	3 months after the operation (^−^x ± s)	Sum	*F*	*P*
Operation group (*n* = 10)	Length of limb shortening neck dry angle	2.49 ± 0.77 101.50 ± 8.09	1.18 ± 0.50 121.00 ± 4.00	1.26 ± 0.53 120.00 ± 3.83	1.64 ± 0.85 114.17 ± 10.63	14.488 37.660	0.000 0.000
Non-operation group (*n* = 12)	Length of limb shortening neck dry angle	2.08 ± 0.70 104.83 ± 7.49	2.08 ± 0.70 104.83 ± 7.49	2.29 ± 0.63 101.17 ± 6.31	2.15 ± 0.67 103.61 ± 7.13	0.409 1.061	0.668 0.358
Sum (*n* = 22)	Length of limb shortening neck dry angle	2.21 ± 0.73 103.81 ± 7.60	1.70 ± 0.77 112.00 ± 10.42	1.83 ± 0.79 109.48 ± 11.12		35.922 46.173	0.000 0.000
*t*	Length of limb shortening neck dry angle	1.323 1.002	3.373 6.452	4.134 8.612	3.630 16.564	*F* = 60.173 *F* = 95.068	
*P*	Length of limb shortening neck dry angle	0.201 0.328	0.003 0.000	0.001 0.000	0.071 0.001	*P* = 0.000 *P* = 0.000

Limb shortening length and collodiaphysial angle of patients were compared with those in the non-operation group in 6 months after the operation. The length of limb shortening in the operation group was higher before the operation and decreased after the operation, increased slightly in 3 months and 6 months after the operation. Collodiaphysial angle was reduced before the operation, increased after the operation, and decreased slightly in 3 months and 6 months after the operation. There was a significant difference in the limb shortening length and collodiaphysial angle among different time points. While no significant difference in the limb shortening length and collodiaphysial angle was found in the non-operation group among different time points before and after surgery. For all time points, except for two groups of patients before operation, there was a significant difference in the limb shortening length and collodiaphysial angle between the operation group and non-operation group in other time points. The length of limb shortening and the neck dry angle of the patients in the 6 months after operation were compared with those in the non-operation group were seen in Table 5.

**Table 5:** The length of limb shortening and the neck dry angle of the patients in the 6 months after operation were compared with those in the non-operation group (*n* = 21)

Groups	Indicators	Before operation (^−^*x* ± *s*)	After Operation (^−^x ± s)	3 months after the operation (^−^*x* ± *s*)	6 months after the operation (^−^*x* ± *s*)	Sum	*F*	*P*
Operation group (*n* = 9)	Length of limb shortening neck dry angle	2.40 ± 0.76 102.44 ± 7.97	1.19 ± 0.53 121.56 ± 3.81	1.21 ± 0.53 120.56 ± 3.61	1.27 ± 0.51 117.00 ± 2.24	1.52 ± 0.77 115.39 ± 9.08	8.919 29.334	0.000 0.000
Non-operation group (*n* = 12)	Length of limb shortening neck dry angle	2.08 ± 0.70 104.83 ± 7.49	2.08 ± 0.70 104.83 ± 7.49	2.29 ± 0.63 101.17 ± 6.31	2.39 ± 0.60 99.58 ± 5.58	2.11 ± 0.65 102.60 ± 6.95	0.700 1.845	0.557 0.153
Sum (*n* = 21)	Length of limb shortening neck dry angle	2.21 ± 0.73 103.81 ± 7.60	1.70 ± 0.77 112.00 ± 10.42	1.83 ± 0.79 109.48 ± 11.12	1.91 ± 0.79 107.05 ± 9.86		35.922 46.173	0.000 0.000
*t*	Length of limb shortening neck dry angle	1.016 0.704	3.162 6.667	4.165 8.233	4.471 9.808	6.609 27.084	*F* = 60.809 *F* = 71.327	
*P*	Length of limb shortening neck dry angle	0.322 0.490	0.005 0.000	0.001 0.000	0.000 0.000	0.019 0.001	*P* = 0.000 *P* = 0.000	

## Discussion

Femoral intertrochanter fracture is a kind of fracture that occurs at the basilar part of the femoral neck and the area within 5 cm distance below the femoral lesser trochanter. It is an extracapsular fracture of the hip joint and could occur at any age of adults, especially the elders [10]. The hip fracture of youths, mainly cleft or pulverize type, is common due to high energy injury, while hip fracture of elders is more often by low energy injury. Femoral trochanter fracture accounts for about 21% of whole-body bone fractures in the elders, with male vs female ratio about 1:3 [11]. Following the aging population, the incidence of intertrochanter fractures increases due to osteoporosis, physical coordination decline, etc. [2, 12]. For most elder patients concurrent with osteoporosis and different degrees of internal medicine diseases, conservative treatment for intertrochanter fractures costs a long time on bed, and it could easily lead to cardiovascular diseases, hypostatic pneumonia, deep vein thrombosis, bedsore, and other complications, with mortality as high as 15–20% [10, 13]. Therefore, surgical treatment is often performed for intertrochanter fractures except for a few patients with worse conditions.

Since its application by Callender in the USA in 1967, DHS has become the standard internal fixation method for femoral trochanter fractures, which is the most representative treatment of the intertrochanter fractures at present [1, 14]. DHS is composed of compressed screws with sliders and side plates with sliding-grooved sockets. The upper part fixes the proximal fractures by the lag screws on the neck of the femur, to extend the length of proximal fractures and effectively hold them. The other end fixes the far end of the fracture with a steel plate structure, both of which are connected through the sleeve, and the stress borne by the femur is transmitted to the solid bone cortex in the middle of the femur to prevent the internal turnover of the hip [15–17]. The nail-plate structure of DHS conforms to the requirements of the hip biomechanics, which has a dual role both in the static and dynamic pressure, making it strongly fixed, so that patients could have quadriceps-femoris-muscle contraction and ankle exercise quite right in the early period post-operation [18, 19]. What is more, less interference will the internal fixation make in the bone marrow cavity, reducing the occurrence of complications such as fat embolism. In addition, the DHS is a kind of internal fixation device made of titanium alloy, the elastic modulus of which is close to the bone tissues, bringing good histocompatibility and high ability to resist fatigue and corrosion. It could be kept in the human body for a long time and is one of the first-choice internal fixator for clinical treatment of ITF at present [2, 20].

The sliding screws and side plates of the DHS internal fixator are firmly fixed to the proximal end of the fracture with strong flexural strength, better medial support effect, thus being positive for hip fracture treatment [3, 4, 21]. However, in clinical application, concerning the effect of patients’ ages, fracture types, reduction effects, and fixed positions, DHS fixation might bring complications such as screws breaking through the femoral head, screws cutting femoral head, coxa vara, screws loosening, and steel plates pulling out [5, 22–24]. It is generally believed that DHS is suitable for the treatment of stable ITF. But for the comminuted unstable ITF, DHS fixation is prone to bringing screws cutting down the femoral head, which leads to complications such as fracture non-union or malunion [25]. According to the literature, the failure rate of the DHS internal fixation in the unstable ITF was as high as 10 to 16%, and could be 24% to 56% especially in reverse ITF [19, 26].

Causes of femoral head cutting in DHS fixation were as follows: (1) the defects of DHS: because of the single screw fixation, DHS had poor rotational resistance, which is easy to cause screw loosening due to shear force and rotation generated by hip activity, resulting in the femoral head cutting. In addition, DHS is a kind of extramedullary fixation. The distance between the femoral head and the steel plate is long. Due to the long arm force and large bending distance, it was easy to cause complications such as fatigue fracture and exit of screws and steel plates [22, 27]. In 11 failure cases of the operation group, 2 cases were caused by the lack of rotational resistance which femoral head cutting occurs. At present, we have partially improved the operative way of DHS for the treatment of ITF. A 7.3-mm hollow nail was added above the fixed screw in DHS to increase the rotational resistance and prevent screw loosening. (2) Injury of ITF itself: because of the deossification in the posteromedial femoral neck, the compressive stress was difficult to conduct through the calcar femorale. Besides, the compressive stress increases 5 to 10 times when the DHS fixed, easily lead to femoral head cutting. Furthermore, the fracture line was parallel to the pulling force when ITF happens, which means no stress support was provided when DHS fixed, making the fracture easy to shift [23, 28, 29]. Four cases from the 11 failure cases that had femoral head cutting had ITF. (3) Improper location or length of screws: the weak cavity formed under the junction of tension line and pressure line of femoral head and neck was called Ward triangle. It was mostly filled with fat tissue in the elderly due to osteoporosis [13]. If the fixed position of the screw is on the upper part, the screw was easily cut after bearing weight, which led to the nailhead pouch out of the femoral head. If the length of the screw was too short, the nail tail could be placed in the Ward triangle, not being fixed under the femoral head cartilage to play an effective role in pressure fixation, and it was also easy to cut femoral head [30–33]. In the 11 failure cases of the operation group, 4 cases of screw position were on the upper part, and 3 cases of screws were too short. Aicale R’s group confirmed that to avoid the risk of mobilization of the cephalic screw and possible subsequent failure of the construct, surgeons should strive for a tip-apex distance (TAD) and calcar referenced TAD (CalTAD) less than 25 mm and the sum of both (TADcalTAD) less than 50 mm when using intramedullary fixation [Aicale, 2018 #58]. (4) Insufficient replacement in the operation: insufficient replacement could lead to small size in collodiaphyseal angle, increasing the shear force the screw suffered and causing the cutting of the femoral head easily [22, 34]. There were four cases with a small collodiaphyseal angle in the 11 failure cases of the operating group. (5) The improper postoperative care [35]: in 11 failed cases of operation group, 3 patients fixed unsuccessfully for walking without crutches after 3 months lacking the agreement of the doctor. (6) Patients themselves: some sicknesses like osteoporosis and chronic diseases in the heart, brain, lungs, etc. in the old people affected the fracture healing [34].

The treatment of the cutting of the femoral head after DHS fixation has been a difficult clinical problem. We used autogenous fibula graft and hollow needle fixation to treat 11 cases of the femoral head cutting after the DHS fixation. The Harris hip joint score was improved after the operation (55.6% was good and excellent in function after the operation, and 77.8% was higher than 70 scores). The shortening length of limb and the change of the neck-shaft angle were significantly improved after operation compared with that before the operation. The shortening of the limb length and the change of the neck-shaft angle of the patients who were operated 3 and 6 months ago were slightly more serious than that before operation by virtue of the weight-bearing activities of the affected limb. But the curative effect was better than that of the patients without the operation. The advantages of the surgical method were those: (1) firm resetting and fixation: the DHS screw was thick, and the nail canal occupied a larger part of the femoral neck so that the intramedullary screws such as pen failed to be used for fixation. Fibula graft could play a role in resisting shearing force. Hollow needles with a smaller diameter (7.9 mm) were used to strengthen the fixation strength. The affected limbs were protected after operation by utilizing lateral, adduction and flexion external rotation position [36]. (2) Filling the cavity of the femoral neck nail canal: after the DHS screw was taken out, the femoral neck was left with a nail canal between 10 and 12 mm, which caused the bone defect, influencing the fracture healing and the fixation strength. The fibula could fill the femoral neck cavity and enhance the stability of fixation when the diameter of the fibula was the same as that of the nail canal after proper pruning. In addition, the bone cortex of the fibula was firm, and the cavity could be immediately provided with strong support after local fixation of it. The fibula was integrated with the surrounding bone tissue in the healing via bone remodeling, and the bone debris generated by the fibula graft could fill the small gap between the surrounding of the fibula and the cancellous bone, eliminating the dead space, and strengthening the repairing ability of the femoral head [37]. (3) Shortening the healing time, promoting early functional exercise: using autogenous fibula graft, bone marrow stromal stem cells and cytokines contained in living fibular bone marrow cavity could differentiate into bone cells, cartilage cells, and vascular endothelial cells, promote fracture healing, shorten healing time, and reduce the incidence of delayed union or non-union. The patients of the operation group had a clinical healing time of 5–8 months (mean 6.8 months). In our department, a neutral fixed frame with an abduction position of the affected limbs was used for 2 weeks after the operation, and CPM function exercise was feasible 4 weeks after the operation. (4) Reduction of femoral head necrosis: the failure of DHS fixation led to femoral head cutting, which was prone to cause the necrosis of femoral head due to the severe damage of femoral head and blood transportation. Fibula graft could be used to fill the cavity of the femoral head and promote its repairment. After transplantation, the graft could reptile, replace and repair, and integrate with the surrounding tissue to heal and reduce the incidence of necrosis of the femoral head. In the 11 patients of the operation group, the fractures of 2 patients failed to heal completely, 1 of which suffered from femoral head necrosis for the patient had severe osteoporosis and various chronic diseases, affecting the fracture healing. (5) Exerting little effect on the function of the lower limbs: fibula graft is chosen to cut out the middle part of the fibula, the length of which was about 8–10 cm. Consequently, the function of the lower limbs was not affected after the operation. When callus was formed 12 weeks later, part of the weight-bearing exercise could be taken part in. When fracture healed 12 months later, they could undergo weight-bearing walk without sticks and the shanks had no motor dysfunction. (6) Short length of hospital stay and low cost: the treatment of femoral head cutting with artificial femoral head replacement is good, but some patients need to change the femoral head. After the application of autogenous fibula graft and hollow needle fixation, the length of hospital stay was shorter, the cost was lower, and the fracture healed completely without reoperation.

## Conclusion

In conclusion, the application of the autogenous fibula graft and hollow nail fixation was effective in treating femoral head cutting after DHS fixation, and patients’ subjective evaluation and objective indicators’ outcomes of follow up were satisfactory, which was worthy of clinical application. However, when choosing operative methods, age and chronic diseases should be considered comprehensively. The long-term effects of this operative method and the comparisons with other methods’ advantages and disadvantages still need further clinical follow-up study and statistical analysis.

## Data Availability

The datasets used and/or analyzed during the current study are available from the corresponding author on reasonable request.

## Abbreviations

DHS: Dynamic hip screw fixation
ITF: Intertrochanteric fractures
